# Gleanings from Our Exchanges

**Published:** 1873-05-01

**Authors:** 


### Gleanings from Our Exchanges.

### ON THE USE OF PANCREATIC EMULSION IN THE WASTING DISEASES OF CHILDREN.

Horace Dobell, M.D. (*Practitioner*, October, 1872), proposes this remedy for that wretched form of atrophy, debility and marasmus in children, where every part of the body wastes away except the abdomen, the state described by Dr. Druitt in his *Vade Mecum* in the following few and graphic words: "Emaciation and voracity; the belly swelled and hard; the skin dry and harsh; the eyes red; the tongue strawberry colored; the breath fœtid; the stools dark-colored and offensive; the bowels sometimes costive, sometimes extremely relaxed; the patient usually dies hectic." The author desires to bring prominently forward that this state, provided there is no advanced lung disease, is rapidly cured by pancreatic emulsion given in doses of a teaspoonful every four hours, and regularly persisted in until fat and flesh are restored. It is, of course, necessary that a proper diet should be insisted upon at the same time; but proper diet without the pancreatic emulsion will not do. In addition to the stress laid upon the influence of the salivary and pancreatic juices upon the digestion of starch, in Dr. Prospero Sonsino's paper, the author says we must not forget the action of the pancreatic juice upon fat; and it is probable that the two functions of the pancreas are sufficiently independent of each other that they may act separately. As shown by experiments, in addition to the action of the pancreas upon fats, it has the power to convert starch into glucin by simple mixture, and this property remains to a certain extent after the pancreas has exhausted its power of acting upon fat. It is possible, therefore, that in different states of depraved health, the one or the other of these properties may be deficient. It is evident that when the power of digesting fat fails to be developed at the proper time, the defect must tell with double force upon children already suffering from deficient digestion of starch.

The children who become the subject of the kind of wasting now spoken of are especially: (1) those suckled by mothers whose milk, though abundant, is extremely deficient in nutritive properties; (2) those brought up by hand; (3) those who, at a later period of childhood, have been subjected to similar chronic defects of diet. It is especially when the mother's milk is poor in fat and lactin that the child becomes dissatisfied and craving; and, in the majority of cases, it is this that first leads to the introduction of farinaceous food, under the popular nursery belief that it is satisfying. As Dr. Sonsino says, if this is given before the power of digesting starch becomes established, of course nothing but mischief can be the result. In the same way that the mother is deprived of fat elements by lactation, so is the child deprived of them by persistency in a diet deficient in milk. The injury is a double one, first by cutting off the supply of fat elements necessary for the protection of the tissues, secondly, by paralyzing the functions of the pancreas by prolonged inactivity. This latter is a point, the author thinks, deserving great attention, and thus accounts in great measure for the impossibility of restoring those ill-nourished, wasted children by any kind of natural diet after they have been allowed to remain in a chronic state of defective nutrition. The author cites three of the very numerous cases where he has seen pancreatic emulsion administered followed by almost magical recoveries. No amount of milk or cream will take the place of the emulsion, the explanation why, notwithstanding milk is also an emulsion of fat, the author thinks turns upon the following points : (1) The fineness of the particles of fat in the pancreatic emulsion ; (2) the permanent character of the molecular mixture ; (3) the fact that different fats in the pancreatic emulsion, consisting, principally, of stearine, margarine and palmatine, have a high melting point, thus differing from the fat of milk, oleine, which has a low melting point.—*Boston Med. & Surg. Journal.*

Puerperal Diseases.—In the last volume of the *Medical Times & Gazette* (July 6, 27, Aug. 24, Oct. 26), Dr. E. Copeman reports a third series of cases of puerperal diseases treated with turpentine. The series includes a variety of conditions coming on after delivery, in all of which, however, the symptoms were more or less indicative of that disease at present known as pnerperal fever. A summary of the cases thus far reported by Dr. Copeman are as follows :—

| | | Deaths. | Recoveries. |
|---|---|---|---|
| 1st series | 21 | 6 | 15 |
| 2d " | 36 | 13 | 23 |
| 3d " | 22 | 5 | 17 |
| | 79 | 24 | 55 |

The third series, which has just been reported, were, as a rule, ushered in with a chill, followed by rapid pulse, with more or less disturbance of the lochia and milk, diarrhœa, tympanites, frequently cerebral symptoms and general depression. The treatment consisted in the use of turpentine fomentations applied over the abdomen, in all cases where the tympanites was marked and accompanied by more or less abdominal pain or tenderness. Turpentine enemata were administered in those cases in which they were not contradicted by pre-existing diarrhœa. In most of the cases, with the exception of those which were accompanied by excessive nausea and vomiting, the *oleum terebinthinæ* was administered internally, in doses varying from half a drachm to a drachm every four hours. Opium was also given to relieve pain and to produce sleep. No bad effects followed the use of the turpentine, but, in almost every case, a marked improvement seemed to follow its administration.

In a subsequent number of the same journal (Nov. 30, 1872), Dr. Copeman gives the account of eleven cases of *phlegmasia dolens* which were successfully treated by warm turpentine fomentations, kept applied constantly to the affected limbs. One of these cases was developed in a non-puerperal woman, while a second occurred in a man.

As regards the nature of puerperal fever, Dr. A. D'Espine has recently contributed a very long article which has been continued through several of the recent numbers of the *Archives Generales de Medicine*, in which he claims that puerperal fever is always the result of an absorption, by means of lacerations and wounds of the utero-vaginal canal, of a greater or less amount of septic material; the severity of the fever depending, in a great measure, on the amount of such material absorbed. The puerperal condition has no special connection with the production of this disease, as a very similar condition is produced in all surgical wards whenever there is a similar absorption of septic material. During the course of this septicæmia, puerperal pyæmia may occur as a complication; but in all such cases, as a rule, it coincides with a suppurative inflammation of the uterine veins. This complication of pyæmia is comparatively rare, and doubtless owes its origin to so-called septic embolisms. He utterly denies the existence of any such disease as milk fever, and claims that the fever which frequently occurs the first seven days after confinement, is almost always due to the re-absorption of a portion of the lochia by the fissures or wounds of the utero-vaginal canal. It is possible for this condition to continue for some weeks, the lochia being in these cases, as a rule, fetid. A close examination will often detect in these cases an ulceration of the cervix uteri or vagina, which are the points of absorption.—*Boston Med. & Surg. Journal.*

Contribution to the Study of Puerperal Septicæmia. — The following is the summary of the conclusions arrived at by M. d'Espine in his long and elaborate articles on this important subject, recently published in the *Archives generales de Medicine.*

1. Puerperal septicæmia consists of a series of symptoms, the gravity of which is in direct relation to the quantity of septic matter absorbed by breaches of surface in the utero-vaginal canal.

2. These symptoms are not peculiar to the puerperal state, and ought to be classed with those produced by septicæmia in the wounded and in animals.

3. The source of puerperal septicæmia is always the uterus or vagina; and all causes which prevent the healing of the bared interior of the uterus, or which favor the production of septic matter in its neighborhood, have an important action in its production.

4. The most common channel of absorption is through the lymphatics, and its passage through them can generally, but not always, be traced by lymphangitis.

5. Peritonitis is the result of the conveyance of septic matters through the lymphatics of the uterus, and it may be compared to the local inflammations which develop round infected wounds.

6. The effect of septic absorbtion is to develop congestions and inflammations in internal organs, chiefly in the lungs, kidneys, and intestines ; subserous ecchymoses and internal interstitial apoplexy ; internal and external inflammations, which localize themselves in the neighborhood of the serous membranes ; *during life*, these actions are recognised by fever, diarrhœa, pulmonary congestion, epistaxis, and often by fugitive cutaneous eruptions.

7. Milk fever has no existence ; febrile action in the first week after delivery almost always depend on absorption of lochia through slight abrasions or lacerations of the utero-vaginal canal. It may continue for some weeks should the uterus not be firmly contracted, or should the lochia be fœtid. In the latter case ulcerations, through which absorption takes place, may almost always be found either on the cervix or in the vagina.

8. These slighter affections are often, but not always, accompanied by angioleucitis and slight perimetritis. When the septic poison continues long we may have consumption and death (*phthisic septique*).

9. Puerperal *pyæmia* is a complication of septicæmia, and is almost always accompanied by the presence of pus in the veins of the uterus.

It is a comparatively rare occurrence, and probably depends on septic embola being impacted in the veins. Metastatic visceral abscesses are secondary to it, while almost all the inflammations of the cellular tissue and of the articulations depend on lymphatic infection, and are not embolic in their origin.—*Medical Record.*

Thermometry of Yellow Fever.—Dr. W. E. Whitehead, U. S. A., has kindly placed in our hands the thermometric charts of seven cases of Yellow Fever, which occurred at the West Bank Quarantine Hospital, N. Y., in the autumn of 1870. We note the following points of interest :

The maximum temperature was invariably reached on the first or second day—mostly on the second—and never mounted so high afterwards, except in one instance.

The maximum ranged from 102° to 104°.

The highest temperature had a direct relation to the severity and prolongation of the disease.

There was one fatal case, and in this the daily range was greater than in any other.

In several instances a tertian movement of temperature was observable.

The defervescence was mostly rapid and irregular, commencing on the third or fourth day.

In two cases the temperature fell to 97° during defervescence, but rose immediately to 98°.

From four to ten days elapsed before the temperature became permanent at the normal standard of 98° ; or, in other words, before convalescence was completely established.

Variability of Pharmaceutic Preparations.—A writer in *The Druggist* reports the result of examinations of eighteen different fluid extracts of belladonna, made by different manufacturers. They ranged from 410 to 80 ; or in other words, the weakest preparation was but one-fifth the strength of the most active. Such facts are startling to practitioners. Doubtless similar uncertainty prevails, though perhaps not to such an extent, in the whole range of pharmacal preparations. A remedy for the evil is imperatively demanded. The responsibility and remedy rest with pharmacists. The rapid progress of pharmaceutical science within a few years past, and the multiplication of associations and schools for its culture, ought to have debarred the possibility of results so embarrassing and disreputable. We are assured that fluid extracts are the most certain and uniform of medicinal preparations, and they are largely prescribed by physicians under this guarantee. We turn the subject over into the hands of our pharmacists for that attention and reform which are alike demanded by the magnitude of the subject in its relation to life and disease, and by their own reputation and their obligations to the community.

Ventilation and Warming.—In a lecture on ventilation, lately delivered before the Franklin Institute, Mr. L. W. Leeds, after detailing the abominations he encountered in his examination of the ventilating arrangements of the Treasury building at Washington, gives the following practical directions concerning provisions for ventilation and warming in the construction of buildings. First, never have long underground fresh-air ducts. Second, never allow a sewer, soil-pipe, foul-air flue, or smoke-flue, to come near the fresh-air supply-flue, for fear of some connection being made between them by carelessness or accident. Third, never heat a building exclusively by currents of warm air. Fourth, always put the heating flues on the outside walls instead of on the inside walls. Fifth, endeavor strenuously to avoid the fresh-air chamber becoming a common receptacle for all the rubbish of a filthy cellar.

On Endocarditis and Embolism.—Sperlling (*Inaugural Dissertation*, Berlin, 1872) analyses 300 cases of endocarditis examined *post mortem* in the Pathological Institute of Berlin during three years, and groups these according to the side of the heart, the valve, or the combination of valves, affected. In regard to embolism, it was found in 29 per cent. of the cases of pure endocarditis; in 26 per cent. the left side was affected, and the right in 2-3 per cent., the lungs being the seat of deposit in the latter cases without exception. In the cases due to endocarditis of the left ventricle, the mitral valves were diseased in 88 per cent., the aortic in 48 per cent.

Contrary to usual acceptation, the kidney was found most often affected with embolism —75 per cent., the spleen being less frequently so—51 per cent.; the brain, 20 per cent.; the liver and intestine, 7 per cent.; the skin, 5 per cent.; the bone-marrow, 3 per cent.; and lastly, the salivary glands and the inner coats of the eye.—*London Med. Record.*

Cerebro-Spinal Meningitis.—This disease is at present prevailing as an epidemic in the Province of New Brunswick. It is at present limited to the neighborhood of Moncton, although a few cases have appeared in St. John; a few cases have also occurred lately in the western part of this Province. It is also prevalent in the Western States, particularly in Kansas. It appears to be very erratic in its course, appearing and disappearing in different parts of the same State or Province, at different times, without seeming to travel in any particular direction. In the cases that have occurred within the past two months, the mortality has been very great. Prof. Loomis, of New York, who has had considerable experience in the treatment of this affection, gives the following :—Sol. saturat-potass bromide, forty minims every two or three hours; quinia sulph., three to five grains every three hours; ice to the head and spine; blisters to the nape of the neck; bleeding, when the constitution of the patient will admit of it, and tonics during convalescence.

The Laryngoscope an American Invention.—Dr. G. Troup Maxwell, of Middletown, Delaware, read before the Delaware State Medical Society, at its last annual meeting, a paper which is published in the New York *Medical Record*, of January 15th, 1873, claiming to have invented and applied the Laryngoscope in 1859, fully four months before the first announcement of the instrument was made by Prof. Czermak. His instrument was constructed by Messrs. Tiemann & Co., of New York, in October, 1859, and the first announcement of Czermak's invention was made in Paris in April, 1860, and in this country in July following. Czermak's invention was prior to that of Dr. Maxwell by a few months, but it could not have been known by the latter, who asserts its originality, but not priority, in the invention, and claims to be the first individual in America to employ the instrument.

On the Use of Quinine in Whooping Cough.—A. Steffen (*Jahrb. fur Kinderheilk.*, N. F. iv. 2 ; *Schmidt's Jahrb*,, 1872, No. 32). Since the autumn of 1868, Steffen has used quinine in various cases of pertussis convulsiva, and generally with good result ; in some cases, on the contrary, there was but slight effect, or none at all. On an average, he gave children between two and five years old, eight to sixteen grains within twenty-four hours. In one case, where the child could not be made to take the medicine, it was used in enema, nine being given in the course of three days, each containing half a drachm. The author reports two cases where the action of quinine seemed quite remarkable.—*Boston Med. & Surg. Journal.*

Influence of Artificial Respiration on the Circulation.—It has been supposed that the mechanical conditions during artificial and natural respiration are so different as to exert very considerable influence on the circulation. In natural inspiration the pressure in the thorax is diminished, while in artificial it is increased.

The result of experiment showed that there was no difference in the effects, provided the artificial injection was not too great. If too much air were introduced, it caused an abnormal elevation of the blood-pressure during inspiration, and an abnormal sinking during expiration. The mean blood-pressure, however, remained unaffected.—*Medical Record.*

Digestion of Starch in Infancy.—(*Gaz. Med. Ital.*, Nov. 1872.)—It has been known that the saliva of newly born animals has not the power of transforming starch into sugar. A recent experimenter has taken the pancreas from kittens and puppies, and has ascertained that the pancreate juice in these animals, when young, is, like the saliva, incapable of converting starch into sugar. The bearing of this fact on the practice of giving starchy food to very young infants is obvious.

Bromide of Iron.—This remedy is advocated by Dr. N. H. Norris, of Beloit, Wisconsin (*Northwestern Medical and Surgical Journal*) as nearly a specific in involuntary seminal emissions and spermatorrhœa. He has administered it three times daily, an hour before or after meals, in doses of from three to five grains, rubbed up in a little syrup. Professor Namais (Practitioner) states that this remedy corrects defective formation of blood, quiets nervous excitation, and produces the combined effect of iron and the bromides. He regards bromide of iron as being in many instances a therapeutic agent of superior value in epilepsy even to the bromide of potassium. — *N. Y. Medical Record.* — *The Southern Medical Record.*

Locomotor Ataxia Treated with Nitrate of Silver.—Prof. Dickerson (*Kansas City Medical Journal*) reports two cases of locomotor ataxia which, following the method of Prof. Wundelich, he successfully treated with nitrate of silver. The medicine was administered in doses of one half-grain, taken after meals. Both patients recovered after about three weeks' treatment. — *The Cincinnati Lancet and Observer.*

Professional Risks. — It was stated in the memoir of M. Guersant, read before the Surgical Society of Paris, that that eminent surgeon contracted syphilis through a wound in his finger received while operating upon a patient affected with that disease, and that his death was the ultimate result of the constitutional infection so produced.

Material for Hypodermic Injections.—The aqueous solutions of the alkaloids are soon impaired by the growth of minute fungi on the surface. This is prevented by the addition of 20 per cent. of glycerin. Sulphuric acid is thought to be better than hydrochloric to facilitate the solution of the salts.

